# Genetic Testing in Differentiated Thyroid Carcinoma: Indications and Clinical Implications

**DOI:** 10.5041/RMMJ.10236

**Published:** 2016-01-28

**Authors:** Sagit Zolotov

**Affiliations:** Institute of Endocrinology, Diabetes, and Metabolism, Rambam Health Care Campus, Haifa, Israel

**Keywords:** Cancer, gene mutation, genetic testing, thyroid

## Abstract

Differentiated thyroid cancer (DTC) is a common and diverse endocrine malignancy. In most patients DTC results in an indolent and curable disease. Nevertheless, disease recurrence rates are relatively high (10%–30%), while 5% of the patients are resistant to conventional treatment and some of these patients are incurable. Over the past 20 years much progress has been made in identifying genetic changes that occur in DTC. In addition, studies aimed to understand the role of these genetic changes in tumorigenesis and their effects on the clinical characteristics of the disease have been conducted. The accrued knowledge has set the stage for development of genetic tests aimed to identify these changes in samples obtained from DTC patients and use this information in the clinical decision process. This paper reviews genetic changes that were identified in DTC, and how the emerging data obtained by genetic testing are currently used to gain key information on the diagnosis, risk stratification, and personalized care of DTC patients.

## INTRODUCTION

The epithelial follicular cell-derived thyroid cancers, papillary thyroid carcinoma (PTC), and follicular thyroid carcinoma (FTC), collectively classified as differentiated thyroid cancer (DTC), account for the majority of thyroid malignancies and are the most common endocrine malignancy.^1^ The follicular cell can also give rise to more aggressive types of cancer such as poorly differentiated thyroid carcinoma (PDTC) and anaplastic thyroid carcinoma (ATC) that are not the focus of this review.

Fine-needle aspiration (FNA) biopsy of thyroid nodules is the gold standard for diagnosing thyroid cancer. Cytology results are classified according to the Bethesda System for Reporting Thyroid Cytopathology.^2^ The Bethesda system recognizes six diagnostic categories and provides an estimation of cancer risk within each category. These categories classify 55%–74% of the thyroid nodules as definitively benign, and 2%–5% of the nodules as definitively malignant.^3^ The remaining samples are cytopathologically inconclusive. Therefore additional studies, or surgical excision, are needed to reach an accurate diagnosis.

Differentiated thyroid cancer carries a favorable prognosis. The overall 10-year survival is 85%.^4^ Mortality is increased especially in patients presenting with extensive local disease or patients with distant metastasis (T4 or M1 according to the American Joint Committee on Cancer TNM staging system for thyroid cancer).^3^,^4^ Standard treatment usually includes primary surgery and thyroid-stimulating hormone (TSH) suppressive therapy, with or without ablation of the thyroid remnant by radioactive iodine (RAI).

Five percent of patients with thyroid cancer have distant metastases recorded at presentation, and 10%–30% of patients have recurrent disease.^1^ Most of these patients, even with an incurable radioiodine-resistant disease, exhibit an indolent course over months or years.^5^

Considerable progress has been made in understanding the molecular mechanisms underpinning DTC in the past 20 years. This progress is best represented by the elucidation of the genetic and epigenetic alterations ultimately affecting key signaling pathways, such as the RAS–RAF–MEK– ERK pathway (MAPK pathway) and the PI3K–AKT– mTOR pathway (PI3K pathway), and their roles in the pathogenesis of DTC.^6^

These signal discoveries have provided an unprecedented opportunity for the identification of diagnostic and prognostic molecular markers that can assist with the diagnosis and treatment of DTC patients. These include developing better tools for the diagnosis of DTC, personalizing the care of DTC patients, and planning treatment protocols based on alterations in transduction signaling pathways and on the biological events arising from genetic changes in DTC.

## EVOLUTION OF DTC

All cancers are thought to share a common pathogenesis based on two cardinal processes: the continuous attainment of random (somatic) mutations and natural selection acting on a transformed clone. Once a single cell acquires a driver mutation that allows autonomous proliferation, the awry clone will grow into a tumor cell population which will invade tissues and metastasize.^7^

**Figure 1.**
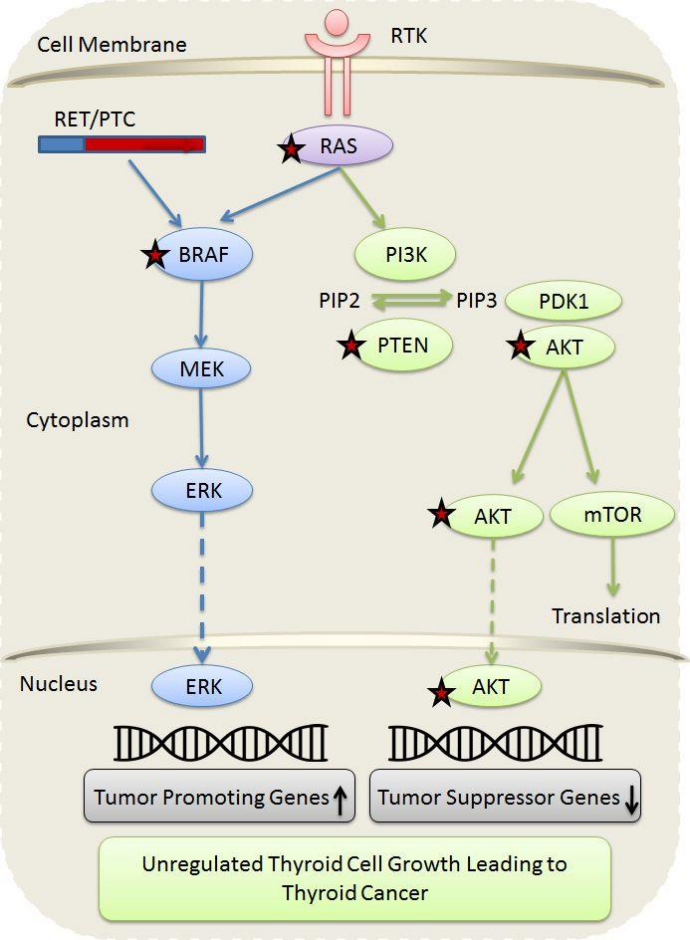
**Genetic Changes Affecting MAPK and PI3K Pathways in Thyroid Cancer.** Somatic or epigenetic changes in genes encoding the components of the MAPK and PI3K pathways (star) lead to constitutive activation of these classical receptor tyrosine kinase (RTK) signaling pathways. These genetic changes include a BRAF mutation, RAS mutation, and RET/PTC gene fusion that constitutively activate the MAPK pathway. Genetic changes that lead to activation of the PI3K pathway include RAS mutation, PTEN mutation or hypermethylation, and AKT1 mutation. Once permanently activated these pathways play a fundamental mechanistic role in thyroid tumorigenesis.

Two key receptor tyrosine kinase (RTK) signaling pathways were found to be affected by somatic mutations in DTC: the MAPK pathway and the PI3K pathway. Once these pathways are constitutively activated by genetic changes such as gene mutations and epigenetic changes, unrestrained cell growth and increased cell survival sustain thyroid tumorigenesis (Figure 1).

## GENETIC CHANGES IDENTIFIED IN DTC

### Gene mutations: BRAF and RAS

The most prevalent and studied mutation in thyroid cancer is the T1799A point mutation of the BRAF gene. This activating gain-of-function mutation results from a thymine to adenine transversion at the nucleotide position 1799, ensuing in the substitution of valine by glutamate at codon 600 in exon 15 (V600E). The unimpaired expression of BRAF^V600E^ (BRAF) mutant protein causes the constitutive activation of the MAPK pathway.^8^ The BRAF mutation occurs in approximately 45% of PTCs. Mutant BRAF is required to sustain tumor growth and is associated with poor clinical outcome as detailed below. Some thyroid nodules have been found to exhibit intra-tumor heterogeneity of the BRAF genotype, with a minority of cells harboring the BRAF mutation while the majority of cells express the wild-type BRAF (BRAF_22_).^9^

Second in prevalence in thyroid cancer are RAS mutations. Mutant RAS proteins lose their intrinsic GTPase activity. These proteins bind GTP but are unable to execute their GTPase activity by hydrolyzing GTP to GDP. Thereby the mutant RAS proteins are “frozen” in a permanent, constitutive “on” state, irrelevant of whether a cognate ligand is around or not.

There are three isoforms of RAS: HRAS, KRAS, and NRAS, the last isoform accounting for the predominant RAS mutation in DTC.^10^ Although RAS is a well-recognized dual activator of the MAPK and PI3K pathways, RAS mutations in thyroid tumorigenesis seem preferentially to activate the PI3K– AKT pathway. A RAS mutation was found in 30%– 45% of FTC patients, in 30%–45% of follicular variant PTC, and in 25% of patients with follicular thyroid adenoma (FTA), which is a benign lesion. The evidence to consider FTA as a premalignant lesion is currently insufficient.^11^

### Gene Translocations: RET/PTC and PAX8/PPARγ

Chromosome translocations are the result of rearrangement of parts between non-homologous chromosomes. The RET/PTC oncogene was first reported in 1987 after DNA extracted from the tumors of five PTC patients induced transforming foci when transfected onto the murine NIH3T3 fibroblast tissue culture cells.^12^ The oncogene was cloned 3 years later and was found to be a chimeric gene generated by the fusion of the RET tyrosine kinase domain (RTK) with the 5’ terminal region of a new gene named H4. In the following years, at least 10 types of RET/PTC variants have been isolated, and among these RET/PTC1 (CCDC6; also known as H4) and RET/PTC3 (NCOA4; also known as ELE1) were identified in DTC. The RET/PTC rearrangement results in ligand-independent dimerization and constitutive RET tyrosine kinase activity. These molecular changes represent an early event in the process of thyroid carcinogenesis and play a critical role in the generation of the PTC and Hurthle cell tumors. In addition, the incidence of RET/PTC rearrangement was shown to be increased in thyroid tumors that developed after radiation exposure.^13^

The paired box 8 (PAX8)–peroxisome proliferator-activated receptor-γ (PPARγ) fusion gene (PAX8/PPARγ) is another prominent recombinant oncogene in thyroid cancer, occurring in up to 60% of FTC and follicular variant PTC (FVPTC). The PAX8/PPARγ gene also occurs in FTA; PAX8/PPARγ exerts a dominant-negative effect on the wild-type tumor suppressor PPARγ and also trans-activates PAX8-responsive genes.^14^

### Gene Amplifications and Copy-number Gains

These genetic alterations cause the replication of a specific genomic sequence leading to increased copies of a gene. This was shown to be the case for genes encoding PI3K pathway components, including PIK3CA, PIK3CB, 3-phosphoinositide-dependent protein kinase 1 (PDPK1; also known as PDK1), AKT1, and AKT2. Activation of this pathway was found to be related to development of FTA and FTC. Overall, genetic copy-number gain in these genes is more prevalent in ATC than in DTC, suggesting that these genetic changes play an important mechanistic role in the progression of thyroid cancer.^15^

### Aberrant Gene Methylation

Gene methylation is an epigenetic hallmark of human cancer that usually results in silencing of gene expression when occurring in gene promoter regions, as hypermethylated chromatin sites impede the accession of transcription factors to consensus DNA sites. Of note, in DTC the BRAF mutation was found to be associated with hypermethylation of several tumor suppressor genes, including the tissue inhibitor of metalloproteinases 3 (TIMP3), SLC5A8, death-associated protein kinase 1 (DAPK1), and retinoic acid receptor-β (RARB). A recent DNA methylation microarray study revealed broad hypermethylation of genes throughout the genome driven by BRAF signaling in PTC cells.^16^ Interestingly, this study also revealed a large range of genes throughout the genome that, driven by BRAF, became hypomethylated and hence are expressed. Hypermethylation of PTEN, a gene coding for a phosphatase normally involved in controlling the extent and duration of the PI3K activity, results in unabated PI3K activation causing an increase in cell proliferation and a decrease in apoptosis.^17^

### miRNA Expression Profile

MicroRNAs (miRNAs) are small (approximately 22 nucleotides) non-protein-encoding RNAs that post-transcriptionally regulate gene expression via suppression of specific target mRNAs. MicroRNAs circulate in a highly stable, cell-free form in the blood, and they can be detected in the plasma and serum. Tumor cells have been shown to release miRNAs into the circulation, and miRNA profiles in plasma and serum have been found to be altered in some cancers and in other diseases. MicroRNA expression in surgical specimen obtained from several human cancers, including DTC, enabled the distinction of benign tissues and malignant samples. In serum samples obtained from patients with thyroid nodules miRNA95 and miRNA190 were shown to be differentially expressed in patients with PTC when compared to patients with benign nodules. This difference in specific miRNAs expression could be adroitly used to identify nodules at high risk for malignancy.^18^

### Genetic Classification of Thyroid Cancers

In a recent study by the “The Cancer Genome Atlas Research Network” tumor samples and matched germline DNA from blood or normal thyroid of patients diagnosed with PTC were used to generate the most extensive data set to date using genome sequencing, genomic and proteomic characterizations including genomic variants, gene expression, and methylation.^19^ This study illustrated several key points in the understanding of the pathogenesis of PTC. These include the mutually exclusive nature of driving somatic genetic alterations in the MAPK and PI3K pathways and the relatively low overall background occurrence of somatic mutations. The study also led to conclusive evidence showing that driver mutations in PTC are clonal events that are present in the majority of cells within the tumor. This is in contrast to thyroid nodules that are not always composed of a homogenous cell population.^9^ Elucidation of the genomic landscape has made the identification of new driver mutations and gene fusions that play a role in the development of DTC (EIF1AX, PPM1D, and CHEK2) possible, leading to the reduction of the fraction of PTC cases without a known driver mutation from 25% to 3%. Further analysis of this data set demonstrated that BRAF mutant PTC signals preferentially through the MAPK pathway and represents a diverse group of tumors, consisting of at least four molecular subtypes with variable degrees of thyroid differentiation. RAS mutant PTCs signal through MAPK and PI3K pathways, resulting in highly differentiated tumors.

## GENOTYPE–PHENOTYPE CORRELATION IN DTC

A BRAF mutation is associated with an aggressive course of DTC. Mutant BRAF carries an increased risk of a more aggressive disease at the time of the diagnosis including extra-thyroidal extension, lymph node metastasis, and advanced tumor stages. A BRAF mutation is also associated with disease recurrence, and in some studies positive BRAF was correlated with increased patient mortality, although evidence is conflicting.^20^ Some of these effects are caused by silencing of thyroid iodide-handling genes, resulting in impairment or loss of radioiodine avidity and hence by the failure of RAI therapy in DTC.^21^ It was also shown that the prevalence of a BRAF mutation is extremely high (around 60%) in recurrent PTC tumors.^20^

The RAS mutations, particularly NRAS mutations, are associated with increased aggressiveness of poorly differentiated thyroid cancer and follicular thyroid cancer, and with decreased patient survival.^22^

Other gene mutations that are considered to be related to the aggressiveness of DTC and their ability to spawn distant metastases are found in PDTC or ATC. Examples are inactivation of the tumor suppressor gene TP53 and an activating mutation of ALK (anaplastic lymphoma kinase) cumulatively promoting tumor progression.^6^

The MAPK and PI3K–AKT pathways are primarily involved in differentiated PTC and FTC, respectively. These alterations are mutually exclusive in well-differentiated thyroid cancer.^23^,^24^ However, simultaneous activation of both pathways becomes more frequent as the grade of thyroid tumors increases. Thus MAPK and PI3K pathway-activating mutations enhance the progression from low grade to high grade of thyroid tumors. For example, mutations in RAS (particularly NRAS) PIK3CA and PTEN, PIK3CA amplification, and hypermethylation of the PTEN promoter increase in frequency from FTA to FTC and to ATC.^25^

The occurrence of the RET/PTC gene rearrangement was found to be a marker for DTC cases associated with radiation exposure. Several studies have tried to associate the presence of RET/PTC rearrangement to clinical parameters with conflicting results. Studies have noted a tendency of the association of RET/PTC activation with lymphatic involvement in otherwise low-risk patients of young age.^26^ In these studies, the RET/PTC rearrangement was associated with lower recurrence rate and improved survival of patients with small tumors. Other investigators did not report any correlation between RET/PTC activation and age, sex, tumor size, staging, number of neoplastic foci, and histological subtype.^12^ The clinical implication of the PAX8/PPARγ translocation, apart from the diagnostic value, is also unknown at the present time.^14^

Genetic copy-number gains are common in genes encoding RTKs and in genes encoding PI3K pathway components such as PIK3CA, PIK3CB, PDPK1, AKT1, and AKT2. These genetic changes are more prevalent in ATC than in DTC, suggesting that these genetic alterations are important for enhancing the aggressive behavior of thyroid cancer cells.^15^

Telomerase reverse transcriptase (TERT) promoter mutations confer enhanced promoter activity in cancer.^27^ These mutations were shown to be associated the presence of other mutations such as BRAF and RAS. The TERT mutations seem to be related to aggressive tumors, presence of distant metastases, and worse response to treatment. In addition, TERT mutations were associated with poor outcome and disease-specific mortality. Furthermore, TERT mutations were not found in benign thyroid lesions.^28^ It was recently shown that the combination of TERT and BRAF mutations in PTC is associated with a more aggressive subtype of PTC compared to tumors carrying BRAF or TERT mutation alone.^29^

## GENETIC TECHNIQUES USED FOR EVALUATION OF DTC

**Figure 2.**
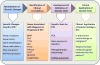
**Development of Genetic Tests for DTC.** The process of development of genetic tests for DTC involves identification of genetic changes that are associated with an aggressive course of DTC, development of a reliable test for identifying these changes from patient samples, and understanding how to apply this knowledge to patient care. The principal aim is to ensure that the use of genetic tests in DTC actually improves patient care as indicated by decreased morbidity of DTC treatment and increased survival of high-risk DTC patients.

The identification of genetic changes thought to have a role in the evolution and progress of DTC— and the attempt to understand their clinical relevance—has set the stage for using molecular markers for the diagnosis and treatment of DTC. The first step in this process was to design a diagnostic platform that is able to identify genetic changes and to report their presence with acceptable validity. Next, an algorithm for the interpretation of large sets of data had to be developed and applied. Further studies were performed to evaluate the ability of the results to improve care of DTC patients. Samples for genetic testing are obtained from cytopathology (FNA) and pathology specimens. Extracted DNA and/or RNA are used for analysis (Figure 2). The currently available methods are discussed below.

## MOLECULAR DIAGNOSTICS IN DTC

The Bethesda classification of thyroid nodule FNA cytology classifies lesions into six categories based on the cytopathological features. Three of the categories carry an increased risk of thyroid malignancy, although they could not provide an accurate diagnosis of DTC. These include: (1) Bethesda category III (atypia of undetermined significance (AUS) and follicular lesion of undetermined significance (FLUS)) that confers a 5%–15% risk of malignancy; (2) Bethesda category IV (follicular neoplasm or suspicious for a follicular neoplasm) that is associated with a 15%–30% risk of malignancy; and (3) Bethesda category V (suspicious for malignancy) with 60%–75% risk of malignancy.^2^

Traditionally these lesions are referred to surgery, and a final diagnosis is provided by the pathology results. It is worth noting that genetic tests could assist in preventing unnecessary surgeries or determine the extent of the surgery in positive cases. Several methods have been applied in an effort to distinguish between benign and malignant nodules.

### Analysis of Gene Mutations and Rearrangements

This test can be performed at local hospital laboratories (rather than sent to a commercial company). Examples of genes that could be interrogated for mutations include BRAF (as a single test) or in combination with RAS, RET/PTC, and PAX8/PPARγ gene rearrangements. The main study in this field was performed by Nikiforov and colleagues.^30^ In this study, tissue samples obtained from FNA were compared to the final pathology results. It was found that measuring frequent DNA abnormalities could be used to “rule in” malignancy while “benignity” could not be determined with high accuracy. The main drawback to the aforementioned study was the relatively large group of samples that were found to be malignant that did not exhibit any of the tested mutations and some benign samples that showed mutations, mostly FTA that were positive for RAS mutations. The BRAF mutation has a very high specificity and positive predictive value for thyroid cancer. The detection of any mutation conferred a high (88%–95%) risk of frank histological malignancy; accordingly, if a given gene mutation is found, further studies or surgery should be pursued.

One example of a commercially available multi-gene panel test is the ThyroSeq assay. This method uses next-generation sequencing technology to detect hundreds of point mutations and gene fusions on more than 60 genes associated with thyroid cancer in samples obtained by FNA. In a recent study^31^ two molecular libraries were used to determine point mutations using isolated DNA and RNA, respectively. The DNA panel included 14 genes. The libraries were mixed together and sequenced using next-generation sequencing. Although the analytic sensitivity of the assay was approximately 1% of mutant alleles, the clinical sensitivity was 5% for BRAF, TP53, AKT1, CTNNB1, PIK3CA, and RET mutations and 10% for NRAS, HRAS, KRAS, PTEN, TSHR, and EIF1AX mutations. In order to avoid false positives, detection of GNAS (G stimulator α-subunit) mutation was considered an indicator of a benign nodule. The method provides both high sensitivity (80% to 90%) and high negative predictive value (∼95%), assuring the patient and the physician that the nodule is likely to be benign.

### Gene Expression Classifier

To perform this assay, a tissue sample obtained by FNA at the local physician’s office is shipped to the Veracyte laboratories in the US, where the sample is processed; RNA is extracted and applied for analysis of 142 transcripts. The report generated after analysis classifies nodules as “suspicious” or “not suspicious.” This information is forwarded to the treating physician who determines the treatment plan to be either watchful waiting or surgical excision. The gene expression classifier (GEC) has a relatively high sensitivity and negative predictive value of 92% and 95%, respectively, for detection of benign nodules; therefore when the GEC result is “non-suspicious for malignancy” watchful waiting could be safely applied. The GEC only identifies approximately 50% of benign nodules (50% specificity), and most nodules classified as “suspicious” by the test will be ultimately diagnosed as benign.^32^

### Mutation Detection and miRNA Expression

In a multicenter study published by Labourier el al.,^33^ surgical specimens and preoperative FNAs that were initially classified as Bethesda category III or IV were tested for 17 gene alterations using the miRInform thyroid test in combination with a 10-miRNA gene expression classifier. In this study mutations were detected in 69% of nodules with malignant outcome. Among mutation-negative specimens, miRNA testing correctly identified 64% of malignant cases and 98% of benign cases. The diagnostic sensitivity and specificity of the combined algorithm were 89% and 85%, respectively. At 32% cancer prevalence in samples classified as Bethesda IV, 61% of the molecular results were benign with a negative predictive value of 94%. Independently of variations in cancer prevalence, the test increased the yield of true benign results by 65% relative to mRNA-based gene expression classification and decreased the rate of avoidable diagnostic surgeries by 69%.

## FUTURE DIRECTIONS USING GENETIC TESTING IN DTC

### Molecular Prognostication

Once DTC is diagnosed by FNA (Bethesda category VI), a decision regarding the extent of the surgery (hemithyroidectomy or total thyroidectomy) has to be made. This decision is usually taken in consideration of clinical risk factors associated with aggressive tumor behavior such as the patient’s age and sex, the size of the initial tumor, and the presence of lymph node and/or distant metastases. Following surgery and determination of the pathological staging,^3^ additional treatment such as radioiodine ablation may be needed, and levothyroxin is administered in order to replace thyroid hormone and to suppress TSH, a follicular cell growth factor. As described above, several genetic mutations are considered markers of aggressive tumor behavior, including mutations in RAS, PIK3CA, PTEN, P53, ALK, and BRAF genes.^6^ Once genetic testing is performed, results should be taken into account in determining the treatment plan and to improve treatment outcome. No prospective studies have been done to address this question. In a multicenter retrospective study of the association of mutant BRAF positivity and mortality in patients with PTC, when analysis of BRAF was adjusted to tumor pathological behavior (patient age, tumor size, the presence of lymph node metastasis, extra-thyroidal extension, distant metastasis, and multifocality), results indicated that the association with BRAF was no longer statistically significant, suggesting that these tumor behaviors are results of the activated underlying pathway.^34^ Considering that the overall mortality in PTC is low and the association is not independent of the pathological tumor features, how to use BRAF and other genetic changes in the management DTC to reduce risk of recurrence and/or mortality risk remains unclear. The greatest utility of BRAF mutation status at this time is in those specific cases where traditional clinicopathological criteria alone would otherwise be unreliable in the assessment of diagnosis, risk stratification, and management. In addition, molecular markers, when used in conjunction with other conventional clinicopathological risk factors, may serve to assist in the risk assessment of PTC as currently incorporated in the American Thyroid Association risk evaluation for structural disease recurrence.^3^

### Molecular Based Treatment

Molecular targeted therapy in DTC should be reserved for patients who have RAI-refractory disease and a documented disease progression according to the Response Evaluation Criteria in Solid Tumor (RECIST), in less than 1 year. Ideally once an initiating oncogenic event is identified molecular targeted therapy could be given based on a strict scientific rationale. To date two drugs, lenvatinib and sorafenib, have been approved by the US Food and Drug Administration for treatment of advanced DTC.^35^ Both drugs are thought to exert their actions by targeting tumor angiogenesis, acting as potent inhibitors of the vascular endothelial growth factor receptors, VEGFR-1, VEGFR-2, and VEGFR-3. These drugs differ in their activity profiles against other kinases that might also contribute to disease pathogenesis. Sorafenib inhibits signaling through the proto-oncogene tyrosine-protein kinase receptor RET, RAF proto-oncogene serine/threonine-protein kinase, and platelet-derived growth factor receptor (PDGFR) β, whereas lenvatinib blocks PDGFRα, RET, mast/stem cell growth factor receptor Kit, and the fibroblast growth factor receptors FGFR-1, FGFR-2, FGFR-3, and FGFR-4.^36^ A careful molecular analysis of the tumor before initiation of treatment may afford a better characterization of the two drugs’ inhibitory effects so that the therapeutic window in which each agent inhibits their molecular targets could be identified.

## CONCLUSION

Accruing information on the molecular pathogenesis of thyroid cancer has opened unprecedented opportunities for the development of novel clinical strategies for the management of DTC. A large amount of data has been collected over the past 20 years regarding molecular changes that occur in DTC. This vast body of information is in the process of being applied to the clinical setting, at the present time with limited success. To improve the utility of genetic testing in DTC treatment further, more studies are needed to assess the validity results, their proper use in the decision-making process, and the overall effect on clinical outcomes. Molecular tests are expensive and not feasible for a large group of patients. Therefore, the information obtained from such tests should be taken into account in combination with more traditional risk factors of DTC ultimately to determine patient care.

## Abbreviations:

ATC: anaplastic thyroid carcinoma
DTC: differentiated thyroid cancer
FNA: fine-needle aspiration
FTA: follicular thyroid adenoma
FTC: follicular thyroid carcinoma
GEC: gene expression classifier
miRNA: microRNA
PDTC: poorly differentiated thyroid carcinoma
PTC: papillary thyroid carcinoma
RTK: receptor tyrosine kinase
TERT: telomerase reverse transcriptase.
